# Magnetic Resonance Spectroscopy and Bipolar Disorder: How Feasible Is This Pairing?

**DOI:** 10.7759/cureus.23690

**Published:** 2022-03-31

**Authors:** Bernardo Sosa-Moscoso, Camila Ullauri, Jose D Chiriboga, Paul Silva, Fernando Haro, Jose E Leon-Rojas

**Affiliations:** 1 School of Medicine, Faculty of Health and Life Sciences, Universidad Internacional del Ecuador, Quito, ECU; 2 Medical Research Department, NeurALL Research Group, Quito, ECU; 3 Radiology, AXXIS Specialty Hospital, Quito, ECU

**Keywords:** mrs, diagnostic imaging, biomarkers, magnetic resonance spectroscopy, bipolar disorder

## Abstract

Bipolar disorder is a psychiatric disorder that affects a significant part of the world's population; however, its diagnosis is difficult, mainly because of the lack of biomarkers and objective tests that aid the clinical evaluation. Proton magnetic resonance spectroscopy (MRS) is a tool that is relatively unused in the medical field. Its application arises from conventional magnetic resonance, and allows non-invasive, *in vivo*, the study of various metabolites and compounds in the human brain. This method may allow the assessment of neurobiochemical alterations in bipolar patients. One of the main advantages of this study type is the simplicity in its use since it only needs a standard magnetic resonator. All these characteristics make it an attractive diagnostic tool that can be used anywhere, including in low-middle-income countries. In conclusion, MRS has potential as a diagnostic tool for bipolar disorder; nevertheless, using it for this purpose still requires additional steps.

## Introduction and background

Bipolar disorder (BD) is a psychiatric disorder with considerable morbidity and mortality. In fact, worldwide BD has a prevalence of 2.4% and represents up to 14% of the total number of suicides [1,2]. In Ecuador, according to the Statistics of Hospital Beds and Discharges of the National Institute of Statistics and Censuses (INEC), in 2019 a prevalence of 6.1 patients with BD was reported for every 10,000 hospitalized patients [3]; Although this figure does not reflect the actual number of people in Ecuador living with BD, it is a potential index that reflects the severe cases that required hospitalization. One of the greatest difficulties of BD is its frequent delayed or mistaken diagnosis since many patients are not diagnosed until the disease has advanced, and many receive erroneous diagnoses such as major depressive disorder (MDD) [4]. Added to this difficulty is the fact that the pathophysiological mechanisms are not very well elucidated, and, consequently, the diagnosis of this disease is purely clinical, without any support from laboratory or imaging tests [5].

On the other hand, magnetic resonance spectroscopy (MRS) is a study derived from traditional magnetic resonance imaging that allows the quantitative analysis of concentrations of different molecules in animal tissues, non-invasively, in vivo; this includes, of course, the human brain [6]. Since its induction in the radiological area, magnetic resonance imaging has become a widely used method of studying the brain and other organs and is available even in developing countries, including Ecuador. Additionally, the use of MRS consists simply of the use of a common magnetic resonator, through the use of pulses and specific sequences [7]. Therefore, it only requires the application of the software that processes the signals captured by 1.5 and 3 Tesla (T) resonators, which are common, albeit in small quantities, in developing countries. This is extremely beneficial since it means that theoretically complex studies can be performed optimally on equipment that is already available to some reference hospitals and private health centers; meaning, we only need to update the software and not the hardware. Specifically, one of the most common MRS methods is based on hydrogen protons: using the same principle of the magnetic spin of hydrogen nuclei, the concentration of different molecules can be detected depending on the number of hydrogen atoms it possesses [7,8].

Our review aims to better characterize the known pathophysiological bases of BD and the changes they generate in brain metabolites, which can be measured by MRS with resonators available in developing countries, that is, of 1.5 or 3 T. Therefore, we will focus only on those metabolites that do not require further spectroscopic processing or state-of-the-art hardware, specifically (Cho), n-acetyl-aspartate (NAA) and those related to glutamate (Glx).

## Review

Pathophysiology of BD, beyond the clinical and non-organic origin

The consensus about BD, as well as about many psychiatric disorders, is that the pathophysiological mechanisms of the disease are not very well elucidated and there is a growing number of studies linking these health problems with defined biological alterations [9]. This paradigm shift is due to the greater variety and effectiveness of laboratory and imaging tests, as well as their increasing availability. In the specific case of BD, a state of chronic neuroinflammation has been identified on several occasions [10], and it has even been suggested that it could be a multisystem inflammatory disorder [11]. Indeed, patients with BD present altered levels of inflammatory markers such as interleukin 4 (IL-4), interleukin 1 receptor antagonist (IL-1Ra), or the soluble interleukin 6 receptor (sIL-6R), changes that reverted when adequate treatment for PD was administered [12,13]. Alterations have even been found in more commonly used markers, such as C-reactive protein, a marker of general inflammation; these disturbances are usually related to stressful life events or psychotic symptoms [14].

**Figure 1:**
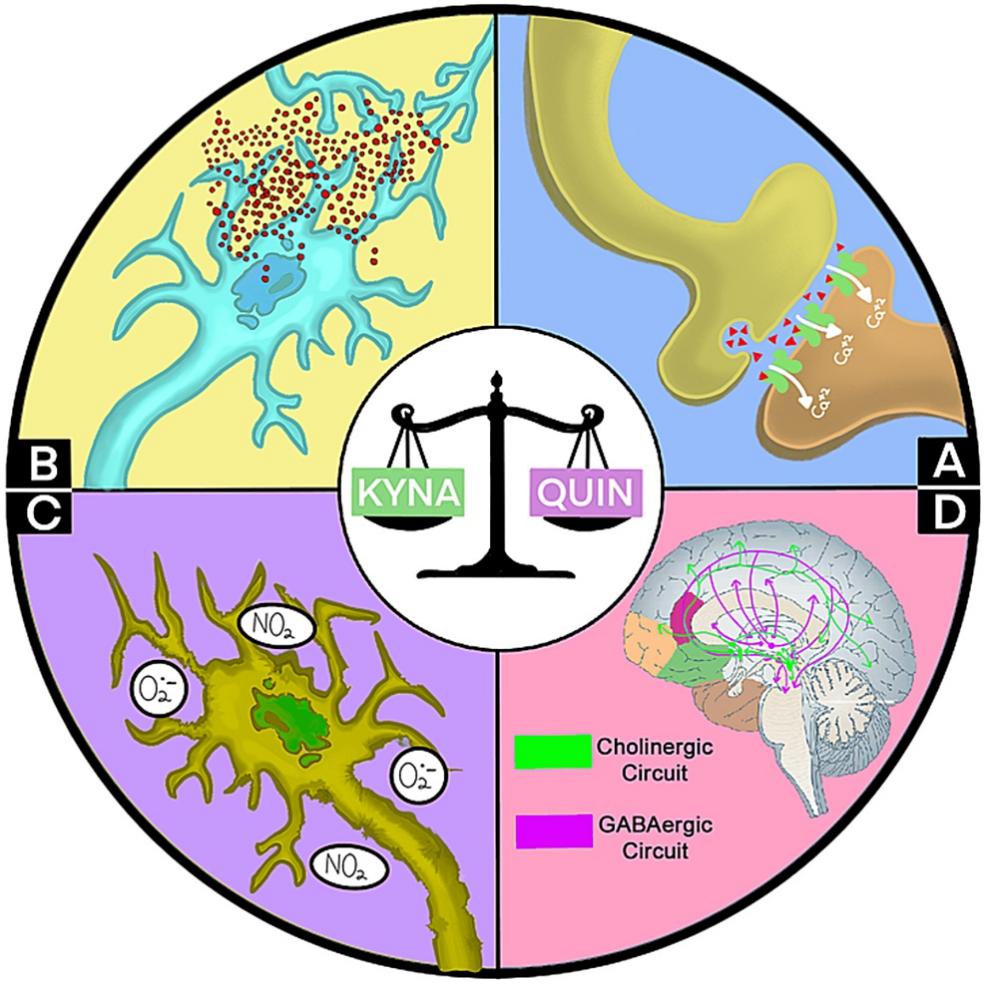
Consequences of the imbalance of the kynurenine pathway Graphical summary of the main consequences of the alteration in the kynurenine pathway. An imbalance between kynurenic acid (KYNA) and quinolinic acid (QUIN) causes a series of alterations: A) Excitotoxicity mediated by N-acetyl-D-aspartate receptors (NMDAr), represented in green, which causes an influx of excessive calcium in the neuron; B) direct neurological damage mediated by the intrinsic cytotoxicity of QUIN; C) production of free nitrogen (NO2) and oxygen (O2-) radicals, which further aggravate neuronal injury; and D) direct alteration of the function of GABAergic and cholinergic circuits. The graphic is an original creation of the authors.

These inflammatory alterations produce several changes in the brain of people with BD, at the cellular and molecular level. Although there are various possible consequences of this chronic neuro-inflammation, such as alterations in mitochondrial metabolism [15] and neurodegeneration [16,17], the most important alteration is that of the kynurenine metabolic pathway. This pathway, under physiological conditions, represents a metabolic mechanism for the degradation of tryptophan, an essential amino acid [18]. In addition, several intermediate metabolites are produced, such as kynurenic acid (KYNA) and quinolinic acid (QUIN). The balance between KYNA and QUIN is delicate, and its alteration results in several deleterious changes for neurons [19]. These changes include those exemplified in Figure 1 [18-20]. All of these alterations could potentially affect key regions of the brain related to memory, cognitive processing, and emotions, and could lead to BD as we know it.

The pairing between BD and MRS

As mentioned above, MRS has the ability to measure the concentration of neurometabolites in the patient's brain; being a non-invasive study and relatively simple to apply, it is promising as a complementary diagnostic tool for pathology that until now has been diagnosed purely by its clinical features. Indeed, several studies have been done analyzing the findings found in BD patients using MRS; the most important findings have been summarized in Table 1.

One of the most important features of MRS is its analysis using voxels, that is, cubic regions of brain tissue of reduced size [6]; through it, the neurometabolites of precise regions of the brain can be analyzed, as well as limiting the amount of cerebrospinal fluid studied or differentiating between gray and white matter. This analysis allows us to derive more specific results regarding the findings in patients with BD.

The findings of BD in MRS are of double importance. First, they mark a point in favor of the pathophysiological process mentioned above; indeed, each molecule analyzed in these studies has a pathophysiological correlation with BD. As mentioned before, there is a process of neuronal damage in BD; thus, molecules such as n-acetyl-aspartate (NAA) and choline (Cho) translate a neurodegenerative process [21] and impaired repair of neuronal membranes, respectively [22]. Consequently, in BD there is a decrease in NAA, reflecting a loss of neuronal integrity [23], and an increase in Cho, which translates into cell membrane damage and altered metabolism of neuronal membranes [24]. Likewise, neurometabolites related to glutamate (glutamate and glutamine, abbreviated as Glx) are directly related to the function and modulation of NMDA receptors [25,26]. As mentioned before, this has a direct correlation with the alteration in the kynurenine pathway. However, the specific findings regarding Glx are mixed; although the most common finding is an increase in its concentration, some studies report a decrease, [27] or no changes at all [26,28,29]. This variation may be due to the region studied, to the different subgroups of BD studied, to an undetermined effect of the medication, or to the limited number of patients studied [26]. Additionally, affected regions such as the anterior cingulate cortex (ACC), prefrontal cortex (PFC), and hippocampus play important roles in cognitive and behavioral ability, memory function, and emotion processing [30-32]. For instance, alterations in the ACC have a negative effect on the ability to adapt and increase the risk of suicide in patients with BD [33,34]. Table 2 summarizes these alterations according to the different brain regions affected.

Second, they represent tangible changes in the brains of patients with psychiatric illnesses and represent an important step toward the future of diagnosing BD, as well as potentially other mental illnesses [35]. Knowing that there are alterations that can be measurable, it is only a matter of being able to determine normal values and ranges, outside of which the diagnosis of PTB or other pathologies would be ratified. Finally, there is the possibility that, by having other diagnostic methods, the definition of BD will be reconsidered [36]. We should remember that the definition and diagnostic criteria for various mental disorders that we use today were proposed by Emil Kraepelin in 1921, as a practical (but imprecise) method for diagnosing psychiatric illnesses such as BD or schizophrenia [37]. This method, which was clearly clinical, consisted of a group of signs and symptoms; however, as they are cognitive and emotional phenomena, their evaluation is quite subjective, which results in reduced diagnostic sensitivity and specificity. Having a better understanding of the pathophysiology, as well as biomarkers that support psychiatric diagnoses, will potentially allow us to discover, in the future, that what we thought were different entities, such as BD and schizophrenia, are actually two phenotypes of the same pathological entity that presents as a spectrum [35,36]. Additionally, having an objective analysis of the severity of the disease and its possible response to drugs (how these metabolites are altered after therapy is started) can help create personalized and more effective treatments tailored to each patient [35,38].

**Table 1:** Metabolite alterations commonly found in cross-sectional MRS studies comparing bipolar patients with healthy controls

Neurometabolite	Disturbance	References
N-acetyl-aspartate	Decreased	Atagun et al. 2018 [28], Borgelt et al. 2019 [27], Bustillo et al. 2019 [39], Port et al. 2008 [40]
Choline	Increased	Cao et al. 2017 [22], Kubo et al. 2017 [41], Michael et al. 2009 [42], Soeiro-de-Souza et al. 2018 [43]
Glutamate – glutamine (GLX)*	Increased	Bustillo et al. 2019 [39], Kubo et al. 2017 [41], Li et al. 2016 [44]
* Given that glutamate and glutamine have very similar chemical compositions, it is difficult to separate their signals in MRS, which is why they are usually reported together (Glx).		

**Table 2:** Regions most affected in bipolar disorder according to altered neurometabolites The regions that are usually affected are the anterior cingulate cortex and the frontal cortex, key brain regions in emotional and cognitive processing. It should be noted that in the case of myo-inositol, studies report an increase in non-alcoholic bipolar patients when compared to alcoholic bipolar patients [51] or in patients who do not consume lithium vs. those who do consume lithium [43].

Neurometabolite	Brain region commonly affected	Disturbance	References
N-acetyl-aspartate	Prefrontal Cortex	Decreased	Hajek et al. 2012 [45], Li et al. 2016 [44], Sassi et al. 2005 [46]
	Anterior cingulate cortex	Decreased	Borgelt et al. 2019 [27], Caetano et al. 2011 [47], Croarkin et al. 2015 [48]
Choline	Anterior cingulate cortex	Increased	Cao et al. 2017 [22], Kubo et al. 2017 [41], Moore et al. 2000 [49]
	Basal Ganglia	Increased	Cao et al. 2016 [24], Howells et al. 2013 [50], Port et al. 2008 [40]
Glutamate-glutamine	Prefrontal Cortex	Increased	Li et al. 2016 [41], Michael et al. 2009 [42], Nery et al. 2010 [51]
Myo-inositol	Frontal Cortex	Increased	Borgelt et al. 2019 [27], Nery et al. 2010 [51], Soeiro-de-Souza et al. 2018 [43]
Table 2.- The regions that are usually affected are the anterior cingulate cortex and the frontal cortex, key brain regions in emotional and cognitive processing. It should be noted that in the case of myo-inositol, studies report an increase in non-alcoholic bipolar patients when compared to alcoholic bipolar patients [51] or in patients who do not consume lithium vs. those who do consume lithium [43].			

## Conclusions

MRS is an effective method in the study of BD. Through this, objective alterations of various metabolites can be found in different key regions of the brain of patients when compared to healthy controls. These findings correspond with the pathophysiological theories attributed to BD, a fact that potentially validates the existence of neuro-biochemical alterations in this psychiatric disorder.

Additionally, the fact that its application only depends on software and a common magnetic resonator, makes it a novel and practical study method that can be applied even in countries with limited resources such as Ecuador. However, certain steps are still needed to validate its use as a diagnostic tool in these patients. Given that most of these studies have been carried out on patients who have previously taken one or several types of medication, the possibility that the findings are biased by the long-term effects of medication cannot be ruled out. A good next step for the use of this diagnostic tool could be a systematic review that validates the findings or long-term prospective studies.
